# 2′-Chloro-4-meth­oxy-3-nitro­benzil

**DOI:** 10.1107/S1600536811019532

**Published:** 2011-05-28

**Authors:** G. Nithya, B. Thanuja, G. Chakkaravarthi, Charles C. Kanagam

**Affiliations:** a Department of Chemistry, Vels Univeristy, Pallavaram, Chennai 600 117, Tamil Nadu, India; b Department of Physics, CPCL Polytechnic College, Chennai 600 068, Tamil Nadu, India; c Department of Chemistry, SRM Valliammai Engineering College, Kattankulathur 603 203, Tamil Nadu, India

## Abstract

In the title compound, C_15_H_10_ClNO_5_, the dihedral angle between the aromatic rings is 87.99 (5)°. The O—C—C—O torsion angle between the two carbonyl units is −119.03 (16)°. The crystal structure is stabilized by a weak intermolecular C—H⋯O hydrogen bond.

## Related literature

For the biological activity of benzil derivatives, see: Mousset *et al.* (2008 ▶); Mahabusarakam *et al.* (2004 ▶); Ganapaty *et al.* (2009 ▶). For bond-length data and related structures, see: Allen *et al.* (1987 ▶); Fun & Kia (2008*a* ▶,*b* ▶).
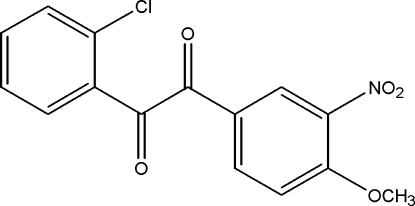


## Experimental

### 

#### Crystal data

C_15_H_10_ClNO_5_
*M* *_r_* = 319.69
Triclinic, 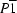

*a* = 7.8559 (2) Å
*b* = 8.1003 (2) Å
*c* = 12.4961 (3) Å
α = 74.893 (1)°
β = 74.809 (2)°
γ = 68.593 (1)°
*V* = 702.32 (3) Å^3^
*Z* = 2
Mo *K*α radiation
μ = 0.30 mm^−1^
*T* = 295 K
0.30 × 0.20 × 0.20 mm

#### Data collection

Bruker Kappa APEXII diffractometer
Absorption correction: multi-scan (*SADABS*; Sheldrick, 1996 ▶) *T*_min_ = 0.917, *T*_max_ = 0.943
17487 measured reflections
3937 independent reflections
3150 reflections with *I* > 2σ(*I*)
*R*_int_ = 0.021

#### Refinement

*R*[*F* ^2^ > 2σ(*F* ^2^)] = 0.042
*wR*(*F* ^2^) = 0.128
*S* = 1.06
3937 reflections
200 parameters
H-atom parameters constrained
Δρ_max_ = 0.48 e Å^−3^
Δρ_min_ = −0.41 e Å^−3^

### 

Data collection: *APEX2* (Bruker, 2004 ▶); cell refinement: *SAINT* (Bruker, 2004 ▶); data reduction: *SAINT*; program(s) used to solve structure: *SHELXS97* (Sheldrick, 2008 ▶); program(s) used to refine structure: *SHELXL97* (Sheldrick, 2008 ▶); molecular graphics: *PLATON* (Spek, 2009 ▶); software used to prepare material for publication: *SHELXL97*.

## Supplementary Material

Crystal structure: contains datablocks I, global. DOI: 10.1107/S1600536811019532/bt5555sup1.cif

Structure factors: contains datablocks I. DOI: 10.1107/S1600536811019532/bt5555Isup2.hkl

Supplementary material file. DOI: 10.1107/S1600536811019532/bt5555Isup3.cml

Additional supplementary materials:  crystallographic information; 3D view; checkCIF report

## Figures and Tables

**Table 1:** Hydrogen-bond geometry (Å, °)

*D*—H⋯*A*	*D*—H	H⋯*A*	*D*⋯*A*	*D*—H⋯*A*
C3—H3⋯O2^i^	0.93	2.53	3.318 (2)	143

Symmetry code: (i) 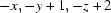.

**Fig. 1.**
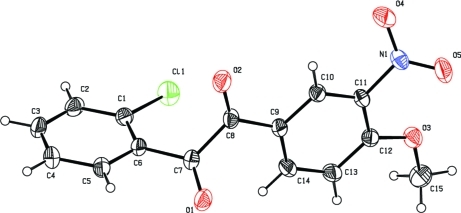
The molecular structure of the title compound, with atom labels and 30% probability displacement ellipsoids for non-H atoms.

## supplementary crystallographic information

### Comment

Benzil derivates exhibit radical scavenging, antibacterial and hypertensive (Mahabusarakam *et al.*, 2004), antiprotozoal (Ganapaty *et al.*, 2009), antiproliferative and antimitotic (Mousset *et al.*, 2008) activities.

The geometric parameters of the title compound (Fig. 1) agree with those in the reported structures (Fun & Kia, 2008*a*,*b*) and the literature values (Allen *et al.*, 1987). The dihedral angle between the two rings is 87.99 (5)°. The mean plane of methoxy and nitro groups are twisted at an angle of 4.95 (8) and 32.19 (6)°, respectively, with the benzene ring (C9—C14).

The dicarbonyl unit has s-*trans* conformation as can be indicated by the torsion angles of O1–C7–C6–C1, and O2–C8–C9–C14 being -145.86 (16) and -171.77 (15)°, respectively. This conformation is authenticated by the torsion angle of O1–C7–C8–O2, being -119.03 (16)°.

The crystal structure exhibit weak C—H···O (Table 1 & Fig. 2) and π···π [*Cg*1···*Cg*1 (-*x*,1 - *y*,2 - *z*) distance of 3.8904 (9)Å and *Cg*2···*Cg*2 (1 - *x*,2 - *y*,1 - *z*) distance of 4.2891 (9) Å; *Cg*1 and *Cg*2 are the centroids of the rings (C1—C6) and (C9—C14), respectively] interactions.

### Experimental

The title compound was synthesized in two steps. The first step involves the benzoin condensation. 4 g of KCN was dissolved in 75cc of water in a one litre flask. To this was added 6.8 g (0.05 mole) of anisaldehyde, 7 g (0.05mole) of 2-chloro benzaldeyde and 75 cc of 95% ethanol. The mixture formed a solution at the boiling temperature and was refluxed for one and half hours. Steam was then passed through the solution until all the alcohol and nearly all the unchanged aldehyde were removed. The condensed water was decanted from the product and later set away to crystallize. The product was then pressed as free as possible from oily material on a suction funnel and washed with cold alcohol. In this way about 9 g of crude product was obtained. The crude mixture was dissolved in hot alcohol and allowed to crystallize slowly. The 2'chloro-4-methoxy benzoin crystallizes out as colourless, hexagonal crystals. From the benzoin about 1 gram was taken and treated with concentrated nitric acid by heating in a water bath inside a fume cupboard for about 3 h until it is free from the smell of nitrogen dioxide. It is then cooled and crystallized using hot ethanol. The obtained benzil is recrystallized using chloroform / acetone in the ratio 3:1. Pure crystals of benzil separates out. The yield is about 70–80%.

### Refinement

H atoms were positioned geometrically and refined using riding model with C—H = 0.93 Å and *U*_iso_(H) = 1.2Ueq(C) for aromatic C—H and C—H = 0.96 Å and *U*_iso_(H) = 1.5Ueq(C) for CH_3_.

### Crystal data

**Table:**

C_15_H_10_ClNO_5_	*Z* = 2
*M**_r_* = 319.69	*F*(000) = 328
Triclinic, *P*1	*D*_x_ = 1.512 Mg m^−^^3^
Hall symbol: -P 1	Mo *K*α radiation, λ = 0.71073 Å
*a* = 7.8559 (2) Å	Cell parameters from 8570 reflections
*b* = 8.1003 (2) Å	θ = 2.7–29.0°
*c* = 12.4961 (3) Å	µ = 0.30 mm^−^^1^
α = 74.893 (1)°	*T* = 295 K
β = 74.809 (2)°	Block, colourless
γ = 68.593 (1)°	0.30 × 0.20 × 0.20 mm
*V* = 702.32 (3) Å^3^	

### Data collection

**Table:**

Bruker Kappa APEXII diffractometer	3937 independent reflections
Radiation source: fine-focus sealed tube	3150 reflections with *I* > 2σ(*I*)
graphite	*R*_int_ = 0.021
ω and φ scans	θ_max_ = 29.6°, θ_min_ = 2.8°
Absorption correction: multi-scan (*SADABS*; Sheldrick, 1996)	*h* = −10→10
*T*_min_ = 0.917, *T*_max_ = 0.943	*k* = −11→10
17487 measured reflections	*l* = −12→17

### Refinement

**Table:**

Refinement on *F*^2^	Primary atom site location: structure-invariant direct methods
Least-squares matrix: full	Secondary atom site location: difference Fourier map
*R*[*F*^2^ > 2σ(*F*^2^)] = 0.042	Hydrogen site location: inferred from neighbouring sites
*wR*(*F*^2^) = 0.128	H-atom parameters constrained
*S* = 1.06	*w* = 1/[σ^2^(*F*_o_^2^) + (0.0635*P*)^2^ + 0.160*P*] where *P* = (*F*_o_^2^ + 2*F*_c_^2^)/3
3937 reflections	(Δ/σ)_max_ < 0.001
200 parameters	Δρ_max_ = 0.48 e Å^−^^3^
0 restraints	Δρ_min_ = −0.41 e Å^−^^3^

### Fractional atomic coordinates and isotropic or equivalent isotropic displacement parameters (Å^2^)

**Table:**

	*x*	*y*	*z*	*U*_iso_*/*U*_eq_	
Cl1	0.15981 (6)	0.62735 (6)	0.72481 (4)	0.06164 (15)	
O1	0.66183 (15)	0.31713 (16)	0.83551 (11)	0.0568 (3)	
O2	0.39794 (18)	0.70912 (17)	0.88704 (10)	0.0583 (3)	
O3	0.82025 (17)	0.96590 (15)	0.40294 (9)	0.0523 (3)	
O4	0.5354 (2)	1.23772 (19)	0.64274 (14)	0.0786 (5)	
O5	0.7972 (2)	1.19529 (19)	0.52797 (13)	0.0714 (4)	
N1	0.6714 (2)	1.14290 (17)	0.58676 (11)	0.0457 (3)	
C1	0.17355 (19)	0.45206 (19)	0.83978 (12)	0.0404 (3)	
C2	0.0214 (2)	0.3912 (2)	0.88416 (15)	0.0505 (4)	
H2	−0.0877	0.4486	0.8552	0.061*	
C3	0.0327 (2)	0.2455 (2)	0.97122 (17)	0.0571 (4)	
H3	−0.0693	0.2046	1.0012	0.069*	
C4	0.1931 (3)	0.1598 (2)	1.01429 (16)	0.0587 (4)	
H4	0.1997	0.0611	1.0730	0.070*	
C5	0.3451 (2)	0.2209 (2)	0.96994 (14)	0.0480 (3)	
H5	0.4537	0.1627	0.9993	0.058*	
C6	0.33717 (18)	0.36820 (18)	0.88208 (12)	0.0376 (3)	
C7	0.50704 (19)	0.4244 (2)	0.83798 (12)	0.0402 (3)	
C8	0.4875 (2)	0.6252 (2)	0.81244 (12)	0.0411 (3)	
C9	0.59015 (19)	0.70248 (18)	0.70630 (12)	0.0384 (3)	
C10	0.59340 (19)	0.87676 (18)	0.69408 (12)	0.0383 (3)	
H10	0.5392	0.9386	0.7540	0.046*	
C11	0.67654 (19)	0.95783 (17)	0.59376 (12)	0.0370 (3)	
C12	0.7569 (2)	0.87131 (19)	0.50031 (12)	0.0397 (3)	
C13	0.7560 (2)	0.6947 (2)	0.51480 (13)	0.0471 (3)	
H13	0.8119	0.6315	0.4556	0.057*	
C14	0.6734 (2)	0.61296 (19)	0.61536 (13)	0.0453 (3)	
H14	0.6732	0.4958	0.6228	0.054*	
C15	0.8938 (3)	0.8816 (3)	0.30568 (15)	0.0650 (5)	
H15A	1.0008	0.7784	0.3192	0.098*	
H15B	0.9292	0.9660	0.2416	0.098*	
H15C	0.8008	0.8441	0.2911	0.098*	

### Atomic displacement parameters (Å^2^)

**Table:**

	*U*^11^	*U*^22^	*U*^33^	*U*^12^	*U*^13^	*U*^23^
Cl1	0.0595 (3)	0.0632 (3)	0.0616 (3)	−0.0207 (2)	−0.0251 (2)	0.0054 (2)
O1	0.0354 (5)	0.0565 (7)	0.0711 (8)	−0.0172 (5)	−0.0089 (5)	0.0038 (6)
O2	0.0625 (7)	0.0654 (7)	0.0542 (7)	−0.0350 (6)	0.0107 (5)	−0.0236 (6)
O3	0.0677 (7)	0.0483 (6)	0.0396 (5)	−0.0255 (5)	0.0027 (5)	−0.0083 (4)
O4	0.0917 (11)	0.0534 (7)	0.0943 (11)	−0.0305 (7)	0.0123 (8)	−0.0384 (8)
O5	0.0858 (10)	0.0603 (8)	0.0791 (9)	−0.0483 (7)	0.0052 (7)	−0.0153 (7)
N1	0.0600 (8)	0.0395 (6)	0.0464 (7)	−0.0238 (6)	−0.0113 (6)	−0.0094 (5)
C1	0.0379 (7)	0.0413 (7)	0.0461 (7)	−0.0148 (6)	−0.0065 (5)	−0.0128 (6)
C2	0.0344 (7)	0.0574 (9)	0.0682 (10)	−0.0180 (6)	−0.0040 (7)	−0.0265 (8)
C3	0.0434 (8)	0.0594 (10)	0.0749 (11)	−0.0309 (7)	0.0117 (8)	−0.0246 (9)
C4	0.0578 (10)	0.0507 (9)	0.0641 (10)	−0.0294 (8)	0.0054 (8)	−0.0036 (8)
C5	0.0434 (7)	0.0443 (8)	0.0538 (9)	−0.0187 (6)	−0.0059 (6)	−0.0013 (6)
C6	0.0344 (6)	0.0384 (6)	0.0425 (7)	−0.0170 (5)	−0.0023 (5)	−0.0088 (5)
C7	0.0368 (7)	0.0461 (7)	0.0403 (7)	−0.0199 (6)	−0.0060 (5)	−0.0035 (6)
C8	0.0387 (7)	0.0463 (7)	0.0448 (7)	−0.0226 (6)	−0.0046 (6)	−0.0090 (6)
C9	0.0385 (6)	0.0377 (7)	0.0431 (7)	−0.0180 (5)	−0.0054 (5)	−0.0080 (5)
C10	0.0406 (7)	0.0397 (7)	0.0398 (7)	−0.0175 (6)	−0.0045 (5)	−0.0122 (5)
C11	0.0419 (7)	0.0333 (6)	0.0414 (7)	−0.0174 (5)	−0.0090 (5)	−0.0070 (5)
C12	0.0412 (7)	0.0403 (7)	0.0388 (7)	−0.0158 (6)	−0.0040 (5)	−0.0084 (5)
C13	0.0570 (9)	0.0404 (7)	0.0447 (8)	−0.0172 (7)	0.0010 (6)	−0.0173 (6)
C14	0.0535 (8)	0.0352 (7)	0.0510 (8)	−0.0191 (6)	−0.0041 (6)	−0.0126 (6)
C15	0.0772 (12)	0.0696 (11)	0.0423 (8)	−0.0258 (10)	0.0097 (8)	−0.0175 (8)

### Geometric parameters (Å, °)

**Table:**

Cl1—C1	1.7315 (16)	C5—H5	0.9300
O1—C7	1.2072 (18)	C6—C7	1.4894 (18)
O2—C8	1.2098 (18)	C7—C8	1.531 (2)
O3—C12	1.3379 (17)	C8—C9	1.4755 (19)
O3—C15	1.4329 (19)	C9—C10	1.3888 (18)
O4—N1	1.2288 (19)	C9—C14	1.392 (2)
O5—N1	1.2081 (18)	C10—C11	1.3727 (19)
N1—C11	1.4649 (17)	C10—H10	0.9300
C1—C2	1.386 (2)	C11—C12	1.4039 (19)
C1—C6	1.387 (2)	C12—C13	1.397 (2)
C2—C3	1.375 (3)	C13—C14	1.375 (2)
C2—H2	0.9300	C13—H13	0.9300
C3—C4	1.371 (3)	C14—H14	0.9300
C3—H3	0.9300	C15—H15A	0.9600
C4—C5	1.386 (2)	C15—H15B	0.9600
C4—H4	0.9300	C15—H15C	0.9600
C5—C6	1.390 (2)		
			
C12—O3—C15	118.41 (13)	O2—C8—C7	116.30 (13)
O5—N1—O4	123.29 (13)	C9—C8—C7	120.11 (12)
O5—N1—C11	119.95 (13)	C10—C9—C14	118.62 (13)
O4—N1—C11	116.76 (13)	C10—C9—C8	118.39 (12)
C2—C1—C6	120.96 (14)	C14—C9—C8	122.86 (12)
C2—C1—Cl1	118.46 (12)	C11—C10—C9	120.09 (12)
C6—C1—Cl1	120.49 (11)	C11—C10—H10	120.0
C3—C2—C1	119.54 (15)	C9—C10—H10	120.0
C3—C2—H2	120.2	C10—C11—C12	122.04 (12)
C1—C2—H2	120.2	C10—C11—N1	116.88 (12)
C4—C3—C2	120.65 (14)	C12—C11—N1	121.04 (12)
C4—C3—H3	119.7	O3—C12—C13	124.79 (13)
C2—C3—H3	119.7	O3—C12—C11	118.04 (12)
C3—C4—C5	119.75 (16)	C13—C12—C11	117.09 (13)
C3—C4—H4	120.1	C14—C13—C12	120.92 (13)
C5—C4—H4	120.1	C14—C13—H13	119.5
C4—C5—C6	120.75 (16)	C12—C13—H13	119.5
C4—C5—H5	119.6	C13—C14—C9	121.19 (13)
C6—C5—H5	119.6	C13—C14—H14	119.4
C1—C6—C5	118.35 (12)	C9—C14—H14	119.4
C1—C6—C7	124.05 (13)	O3—C15—H15A	109.5
C5—C6—C7	117.59 (13)	O3—C15—H15B	109.5
O1—C7—C6	122.20 (13)	H15A—C15—H15B	109.5
O1—C7—C8	117.75 (12)	O3—C15—H15C	109.5
C6—C7—C8	119.31 (12)	H15A—C15—H15C	109.5
O2—C8—C9	123.30 (13)	H15B—C15—H15C	109.5
			
C6—C1—C2—C3	−0.1 (2)	O2—C8—C9—C14	−171.77 (15)
Cl1—C1—C2—C3	176.25 (12)	C7—C8—C9—C14	14.6 (2)
C1—C2—C3—C4	−0.1 (3)	C14—C9—C10—C11	0.4 (2)
C2—C3—C4—C5	0.2 (3)	C8—C9—C10—C11	−175.55 (13)
C3—C4—C5—C6	−0.1 (3)	C9—C10—C11—C12	1.3 (2)
C2—C1—C6—C5	0.3 (2)	C9—C10—C11—N1	178.94 (12)
Cl1—C1—C6—C5	−176.05 (11)	O5—N1—C11—C10	148.61 (15)
C2—C1—C6—C7	179.26 (13)	O4—N1—C11—C10	−31.4 (2)
Cl1—C1—C6—C7	3.0 (2)	O5—N1—C11—C12	−33.7 (2)
C4—C5—C6—C1	−0.2 (2)	O4—N1—C11—C12	146.30 (16)
C4—C5—C6—C7	−179.23 (15)	C15—O3—C12—C13	−0.3 (2)
C1—C6—C7—O1	−145.86 (16)	C15—O3—C12—C11	−176.86 (15)
C5—C6—C7—O1	33.2 (2)	C10—C11—C12—O3	174.20 (14)
C1—C6—C7—C8	44.2 (2)	N1—C11—C12—O3	−3.4 (2)
C5—C6—C7—C8	−136.81 (14)	C10—C11—C12—C13	−2.6 (2)
O1—C7—C8—O2	−119.03 (16)	N1—C11—C12—C13	179.86 (14)
C6—C7—C8—O2	51.37 (19)	O3—C12—C13—C14	−174.26 (15)
O1—C7—C8—C9	55.0 (2)	C11—C12—C13—C14	2.3 (2)
C6—C7—C8—C9	−134.60 (14)	C12—C13—C14—C9	−0.7 (3)
O2—C8—C9—C10	4.0 (2)	C10—C9—C14—C13	−0.6 (2)
C7—C8—C9—C10	−169.64 (13)	C8—C9—C14—C13	175.07 (15)

### Hydrogen-bond geometry (Å, °)

**Table:**

*D*—H···*A*	*D*—H	H···*A*	*D*···*A*	*D*—H···*A*
C3—H3···O2^i^	0.93	2.53	3.318 (2)	143

Symmetry codes: (i) −*x*, −*y*+1, −*z*+2.
